# Configuration–packing synergy enabling integrated crystalline-state RTP and amorphous-state TADF

**DOI:** 10.3762/bjoc.22.16

**Published:** 2026-02-02

**Authors:** Ruiyan Wang, Yunan Wu

**Affiliations:** 1 Sendelta International School, Shenzhen 518038, China; 2 Chongqing Institute of Green and Intelligent Technology, Chinese Academy of Sciences, Chongqing 400014, China; 3 New Materials Research Institute of CASCHEM (Chongqing) Co., Ltd, Chongqing 400714, China

**Keywords:** imide, π–π stacking, room-temperature phosphorescence, thermally activated delayed fluorescence

## Abstract

A twisted D–π–A molecule, PI-Cz **1**, was designed and synthesized using phthalimide as the acceptor, carbazole as the donor, and a phenylene bridge. Single-crystal X-ray diffraction revealed a markedly non-coplanar skeleton. Calculations based on the crystallographic geometry and frontier-orbital analysis indicate that the HOMO and LUMO are localized on the carbazole and phthalimide fragments, respectively, affording a small singlet–triplet energy gap. In the solid state, compound **1** exhibits pronounced phase dependence: powder samples display room-temperature delayed emission with principal bands at 550/600 nm and a lifetime of ≈0.39 s that undergoes strong thermal quenching, diagnostic of room-temperature phosphorescence. In contrast, amorphous films show no RTP; their delayed component grows with temperature and shares the same peak position as the prompt emission, consistent with thermally activated delayed fluorescence (TADF). Correlating temperature-dependent lifetimes with phase characterization indicate that, in amorphous environments lacking ordered π–π stacking and rigid confinement, the small Δ*E*_ST_ promotes reverse intersystem crossing, yielding delayed fluorescence; whereas in powder states, intermolecular interactions enhance spin–orbit coupling and crystallinity suppresses nonradiative decay, thereby activating RTP. This work achieves an integrated “crystalline-state RTP–amorphous-state TADF” regulation within a single molecule.

## Introduction

Organic light-emitting materials have attracted significant attention due to their remarkable potential across a wide array of applications [1–3], such as in display technologies [4], information security and data storage [5], time-resolved biological imaging [6], chemical and biological sensing [7], as well as energy transfer and upconversion processes [8]. The unique characteristics of organic materials, including their solubility, ease of processing, molecular structure programmability, and the tunability of their emission color, lifetime, and efficiency, make them highly versatile for integration into advanced optoelectronic devices [9–11]. The performance limits of these devices, particularly in terms of their brightness, longevity, and efficiency, are largely dependent on the ability to precisely control the dynamics of the excited states, with a particular focus on the generation, migration, and efficient utilization of triplet excitons, which are crucial to device performance.

In pure organic systems, the effective utilization of triplet excitons typically proceeds along two primary pathways: room-temperature phosphorescence (RTP) and thermally activated delayed fluorescence (TADF) [2,12–19]. RTP is a process where the spin-forbidden transition from the triplet state to the ground state becomes observable at room temperature due to the enhancement of spin–orbit coupling (SOC) and suppression of both intra- and intermolecular non-radiative decay pathways [20–22]. Several strategies have been developed to enhance RTP in organic materials, such as incorporating functional groups with n→π* characteristics, such as carbonyl [23], sulfone [24], or sulfonamide groups [25], constructing rigid hydrogen-bonded [26] or ionic networks [13], and utilizing crystallization [27] or confinement environments [28] to restrict molecular motion. These strategies have been implemented in various systems, such as aromatic diketones, diimides, urea/amides self-assembled solids, and carbonyl-containing polymer hosts.

In contrast, TADF relies on the reduction of the singlet–triplet energy gap (Δ*E*_ST_), facilitating thermally activated reverse intersystem crossing (RISC) from the triplet state to the singlet excited state, where the excitation energy is released via delayed fluorescence [29]. Common molecular design approaches for TADF include twisted donor–acceptor structures (for example, carbazole or phenoxazine as donors and triazine, nitrile, or carbonyl groups as acceptors) as well as B/N multiresonance frameworks [30–32]. These strategies have been successful in achieving efficient triplet exciton recovery and enhancing device performance in both solution and solid states.

Although significant progress has been made in developing systematic molecular and solid-state engineering strategies for RTP and TADF, examples of simultaneous “phosphorescence-delayed fluorescence” switching within a single molecule under different environments or phases are still relatively rare [33]. The underlying challenge lies in the different demands that RTP and TADF place on the microscopic configuration and solid-state environment. RTP typically favors molecular rigidity, confinement, and specific intermolecular coupling to enhance SOC and suppress non-radiative decay channels, while TADF relies more on donor–acceptor decoupling and smaller Δ*E*_ST_ to lower the thermal activation barrier for RISC. Thus, a key challenge in the field is how to establish adjustable synergy between the molecular-scale configuration design and material-scale packing/confinement engineering to enable the controlled switching between RTP and TADF.

Among the potential receptor units, imide, with its strong electron-withdrawing nature and prominent n→π* contribution from the two carbonyl groups, offers a dual advantage: it promotes intersystem crossing (ISC) and spin–orbit coupling (SOC) for RTP and, when coupled with carbazole and other donor segments, facilitates the construction of twisted D–A scaffolds that reduce Δ*E*_ST_, making them suitable for TADF [34–36]. On the one hand, the dual carbonyl groups provide an effective pathway for the singlet-to-triplet transition according to the El-Sayed rule, thus enhancing the ISC rate and RTP visibility. On the other hand, imide, when used as an acceptor with carbazole or phenoxazine as donors, forms a small energy gap with spatially separated frontier orbitals, thereby facilitating thermally activated reverse intersystem crossing (RISC) and enabling TADF. Therefore, a molecular platform based on imide is expected to achieve programmable triplet management through a “configuration-decoupling + packing/confinement synergy” strategy. This platform can enable the selective activation of either RTP or TADF channels in different phases, thus contributing to both fundamental mechanistic studies and the expansion of device applications.

In this work, phthalimide (imide) was selected as the acceptor fragment, carbazole as the donor, and a phenylene bridge was introduced to construct a twisted D–π–A molecular backbone. This molecular backbone achieves, on the one hand, frontier orbital spatial decoupling through donor–acceptor separation, reducing Δ*E*_ST_ and enabling TADF activation in amorphous environments. On the other hand, it retains the n→π* characteristics of the carbonyl-containing acceptor and potential intermolecular interactions, providing conditions for enhanced SOC and RTP manifestation in crystalline or partially crystalline states. By combining single-crystal structural analysis, quantum calculations, and steady-state/time-resolved/temperature-dependent spectroscopy, the triplet exciton management and channel selectivity in different phases of PI-Cz **1** were systematically studied, validating the feasibility of “configuration-packing synergy” for controllable RTP–TADF switching within the same molecule.

## Results and Discussion

**Figure 1:**
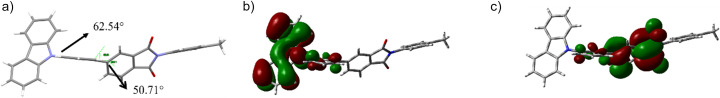
a) Single-crystal structure of **1**, b) HOMO distribution calculated on the crystallographic geometry, and c) LUMO distribution calculated on the crystallographic geometry.

Single crystals of **1** were successfully obtained through the method of slow solvent evaporation, which allows for the formation of high-quality crystals suitable for detailed structural analysis. The crystal structure of **1** was thoroughly characterized using single-crystal X-ray diffraction (CCDC 2492630). This technique provides precise three-dimensional information about the atomic arrangement within the crystal lattice. As depicted in Figure 1a, the crystal structure reveals that the dihedral angle between the phthalimide moiety and the bridging phenyl ring is 50.71°, while the angle between the bridging phenyl ring and the carbazole unit is 62.54°. These significant angles result in a distinctly twisted molecular framework, which plays a crucial role in spatially separating the donor (carbazole) and acceptor (phthalimide) segments of the molecule. This twisting and separation of the donor and acceptor parts of the molecule are critical for influencing the electronic properties and excited-state dynamics of **1**.

Building on the crystallographic data, quantum-chemical calculations were performed using the Gaussian 09 software at the B3LYP level of theory. The calculations revealed that the highest occupied molecular orbital (HOMO) is primarily localized on the carbazole donor unit, while the lowest unoccupied molecular orbital (LUMO) is mainly concentrated on the phthalimide acceptor unit, as illustrated in Figure 1b and 1c. This significant separation of the HOMO and LUMO indicates a substantial frontier-orbital decoupling between the donor and acceptor, which is essential for efficient charge transfer and the functionality of the material in optoelectronic applications.

As shown in Table 1, the calculated excited-state energies indicate that the triplet energy level is 2.7447 eV, and the singlet energy level is 2.7677 eV, which corresponds to a very small singlet–triplet energy gap (Δ*E*_ST_ ≈0.02 eV). Such a narrow Δ*E*_ST_ is a favorable condition for efficient reverse intersystem crossing, a process where the triplet state can be converted to the singlet state under thermal activation. This small Δ*E*_ST_ value suggests that **1** has strong potential to exhibit TADF, which is a process where delayed fluorescence is observed due to thermal activation. Alternatively, the material may also exhibit RTP, depending on the environmental conditions and the specific molecular interactions. These findings highlight the promising photophysical properties of **1**, making it a suitable candidate for use in advanced optoelectronic devices, such as organic light-emitting diodes (OLEDs), where efficient triplet-state management and delayed fluorescence are highly desired.

**Table 1:** Energy-level parameters calculated from the single-crystal geometry of **1**.

Compound	S_1_ (eV)	T_1_ (eV)	Δ*E*_ST_ (eV)	HOMO (eV)	LUMO (eV)	Energy gap (eV)

**1**	2.77	2.74	0.03	−2.61	−5.74	3.13

**Figure 2:**
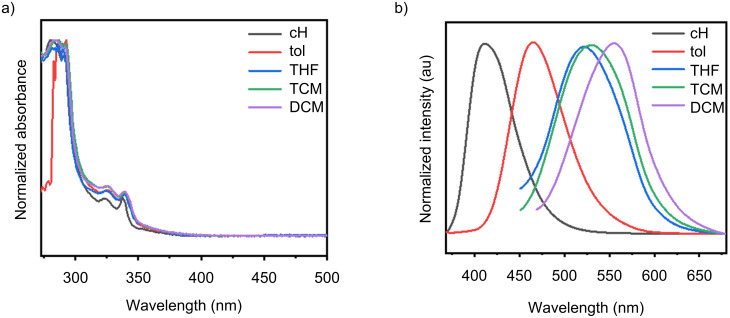
Photophysical properties of **1** in solvents of varying polarity: a) UV–vis absorption spectra and b) fluorescence emission spectra (excitation wavelength: 365 nm).

In different solvents, the UV–vis absorption maxima and band profiles of the compound remain essentially unchanged, whereas its fluorescence emission undergoes a monotonic red shift with increasing solvent polarity (or polarizability), accompanied by an enlarged Stokes shift – a typical solvatochromic behavior. The weak solvent dependence of the absorption spectra indicates that the difference in dipole moment between the ground state and the vertically accessed excited state (Franck–Condon region) is small, and that no pronounced ground-state specific interactions are operative; ground-state-dominated spectral shifts can therefore be largely excluded. By contrast, the strong polarity sensitivity on the emission side points to conformational/electronic density reorganization after excitation, relaxing to an emissive state with a larger dipole moment dominated by charge transfer (CT). As solvent polarity increases, preferential solvation stabilizes this higher-dipole excited state more than the ground state, lowering the excited-state energy and thus the photon energy, which manifests macroscopically as a red-shifted emission. In polar media, the oriented polar solvent shell stabilizes the CT state more effectively than the ground or locally excited (LE) state, further decreasing the emission energy. Consistent with these trends (Figure 2), compound **1** exhibits nearly identical UV–vis absorption across solvents, while its emission shifts bathochromically with increasing polarity, indicating that the luminescence of **1** arises predominantly from intramolecular charge transfer.

**Figure 3:**
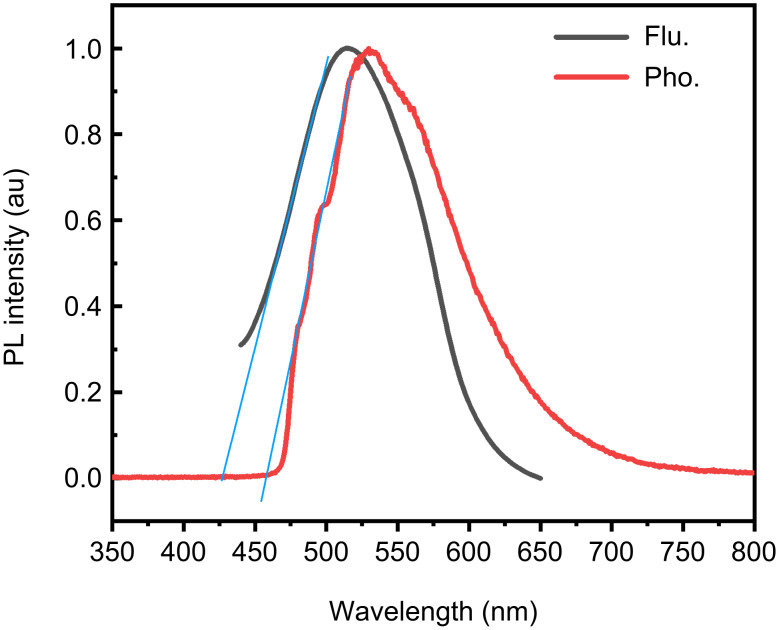
Fluorescence and phosphorescence spectra of **1** in THF (excitation wavelength: 365 nm).

In a dilute tetrahydrofuran (THF) solution at a concentration of 1.0 × 10^−5^ M, the steady-state fluorescence spectrum of **1** was recorded, and its phosphorescence spectrum was subsequently measured at a temperature of 77 K to investigate its low-temperature behavior (Figure 3). Following the established spectroscopic conventions, the Δ*E*_ST_ was estimated by calculating the difference between the onset energies of the fluorescence and phosphorescence bands.

This energy difference was determined using Equation 1:


[1] $$E=\frac{hc}{\lambda }$$

where *E* is the energy, *h* is Planck's constant, *c* is the speed of light, and λ is the wavelength corresponding to the onset of the fluorescence or phosphorescence emission.

From the experimentally determined onset wavelengths, the fluorescence onset (λ_S1,onset_) was found to be 428 nm, while the phosphorescence onset (λ_T1,onset_) was measured at 457 nm. Using these values, the corresponding energies for the singlet and triplet states were calculated as *E*_S1_ = 2.90 eV and *E*_T1_ = 2.71 eV

The energy gap between the singlet and triplet states, Δ*E*_ST_, was then calculated to be 0.18 eV. This narrow singlet–triplet energy gap is a crucial factor in enabling efficient RISC processes, which is an important feature for TADF and RTP. This result provides valuable insights into the excited-state dynamics of **1** and its potential for optoelectronic applications.

**Figure 4:**
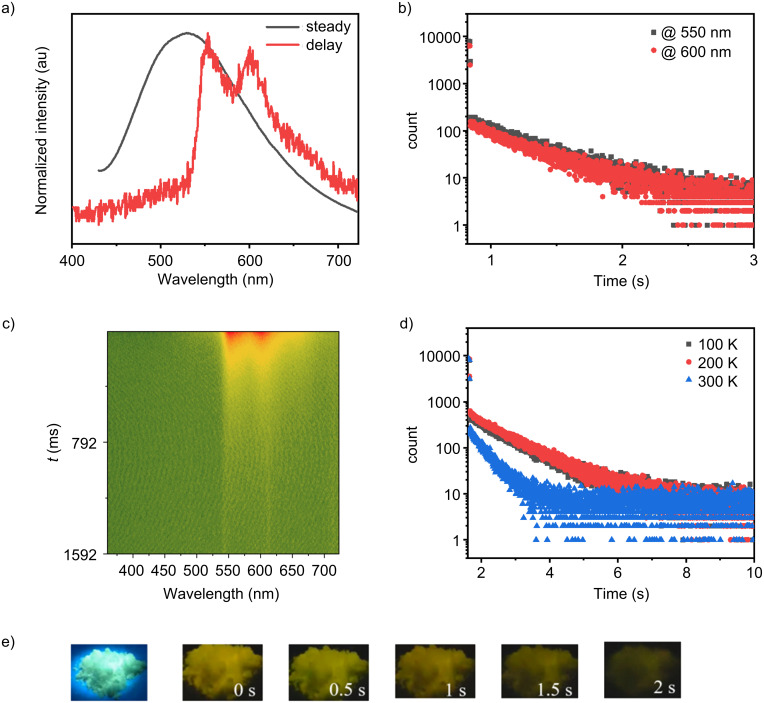
a) Steady-state and delayed emission spectra of **1**, b) room-temperature emission lifetimes monitored at 550 nm and 600 nm, c) time-resolved emission spectra, d) temperature-dependent lifetime at 550 nm and e) photographs of **1** under UV irradiation and after removal of the UV source. All PL spectra and lifetime measurements were recorded with λ_ex_ = 365 nm.

In order to fully characterize the solid-state emission properties of **1**, a comprehensive set of measurements was performed, including steady-state and post-excitation (delayed) emission spectra, as well as decay kinetics. These measurements were conducted using a CCD detector, allowing for precise recording of both fluorescence and phosphorescence behaviors. As shown in Figure 4a, under ultraviolet (UV) excitation, the powder sample of **1** displays a steady-state fluorescence band with its maximum emission at 530 nm, which corresponds to typical fluorescence behavior in this material under the given excitation conditions. Upon turning off the UV light source, the sample continues to emit light, displaying a persistent afterglow that remains visible (Figure 4e). The delayed emission spectrum (Figure 4a) obtained after the UV source is turned off exhibits two main emission bands at 550 nm and 600 nm. Interestingly, the lifetimes of the emissions at these two wavelengths were found to be identical, with a measured value of τ = 0.39 s, and the relative intensity of these two emission bands remains remarkably stable over time (Figure 4c). This consistent intensity ratio over time strongly suggests that both bands originate from the same emitting center within the material, most likely representing different vibronic or microenvironmental emissions from a common triplet state.

To further investigate the emission dynamics, temperature-dependent lifetime measurements were conducted, which revealed a clear and significant reduction in the emission lifetime as the temperature increased (Figure 4d). This observation supports the hypothesis that the delayed emission is a result of RTP, as the sub-second lifetime and the pronounced thermal quenching behavior are characteristic features of RTP. Additionally, the red shift observed in the delayed emission spectrum compared to the steady-state emission is consistent with the behavior of lower-energy emissions that are dominated by triplet states. Overall, these results demonstrate that **1** efficiently generates triplet excitons in the powder state which radiatively deactivate through phosphorescence. This behavior provides a solid foundation for further studies aimed at understanding the triplet-related photophysics of **1** and exploring its potential applications in various optoelectronic devices. The ability to control and utilize both fluorescence and phosphorescence in **1** opens up promising avenues for future research, particularly in the development of advanced materials for light-emitting devices.

**Figure 5:**
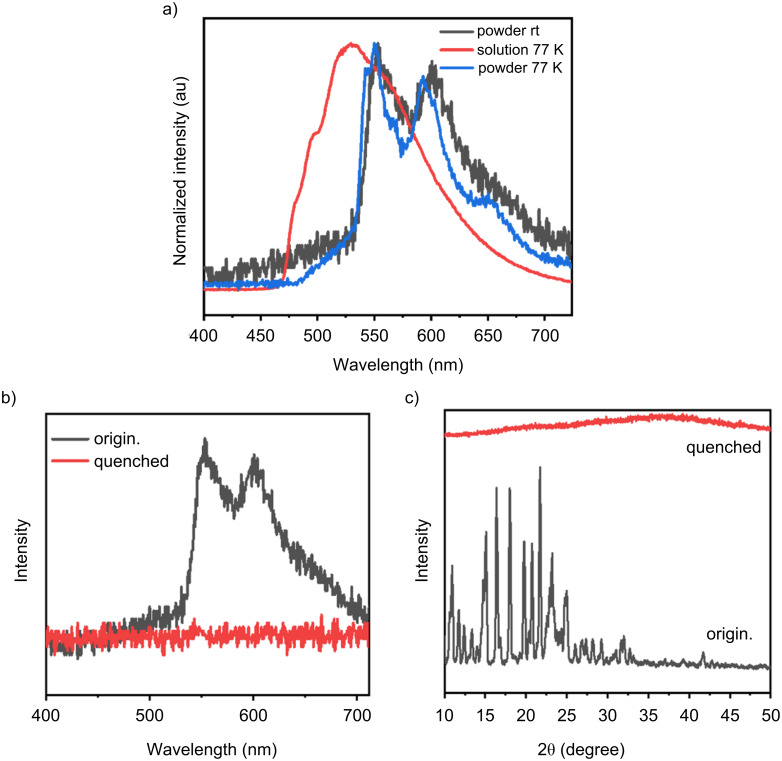
a) Phosphorescence spectra of **1** in the powder state at room temperature and at 77 K, and in dilute solution at 77 K (excitation wavelength: 365 nm), b) phosphorescence spectra of **1** powder before and after melt quenching (excitation wavelength: 365 nm), and c) powder X-ray diffraction (PXRD) patterns of the powder and the melt-quenched solid.

At room temperature, phosphorescence was not observed in the dilute solution of the compound. To further investigate the emission behavior, the phosphorescence spectra were compared between the dilute solution at 77 K and the powder form of the material, as shown in Figure 5a. A significant red shift in the phosphorescence emission was observed for the powder compared to the solution, suggesting that the RTP in the powder phase is influenced by intermolecular packing effects. This observation implies that the organization and interaction between molecules in the solid state play a crucial role in facilitating the phosphorescence emission at room temperature.

To further explore this hypothesis, the powder was subjected to heating to its melting point followed by rapid quenching with liquid nitrogen. The resulting solid, after this treatment, was found to show no RTP when examined at room temperature, as demonstrated in Figure 5b. This indicates that the specific molecular arrangement necessary for RTP was disrupted upon melting and subsequent rapid cooling, suggesting that the ordered structure in the solid phase is essential for the RTP phenomenon.

In addition to the optical measurements, powder X-ray diffraction analysis was conducted to examine the crystalline structure of both the as-prepared powder and the melt-quenched sample (Figure 5c). The diffraction pattern of the as-prepared powder showed the presence of a certain degree of crystallinity, indicating that the molecules were organized in a regular, ordered manner. However, the diffraction pattern of the melt-quenched sample revealed an amorphous nature, lacking the sharp peaks associated with crystalline materials. These findings collectively support the conclusion that the manifestation of RTP is not solely dependent on molecular aggregation but also on the presence of a specific crystalline packing motif that allows for effective intermolecular interactions and the stabilization of triplet excitons, which are necessary for phosphorescence to occur at room temperature.

**Figure 6:**
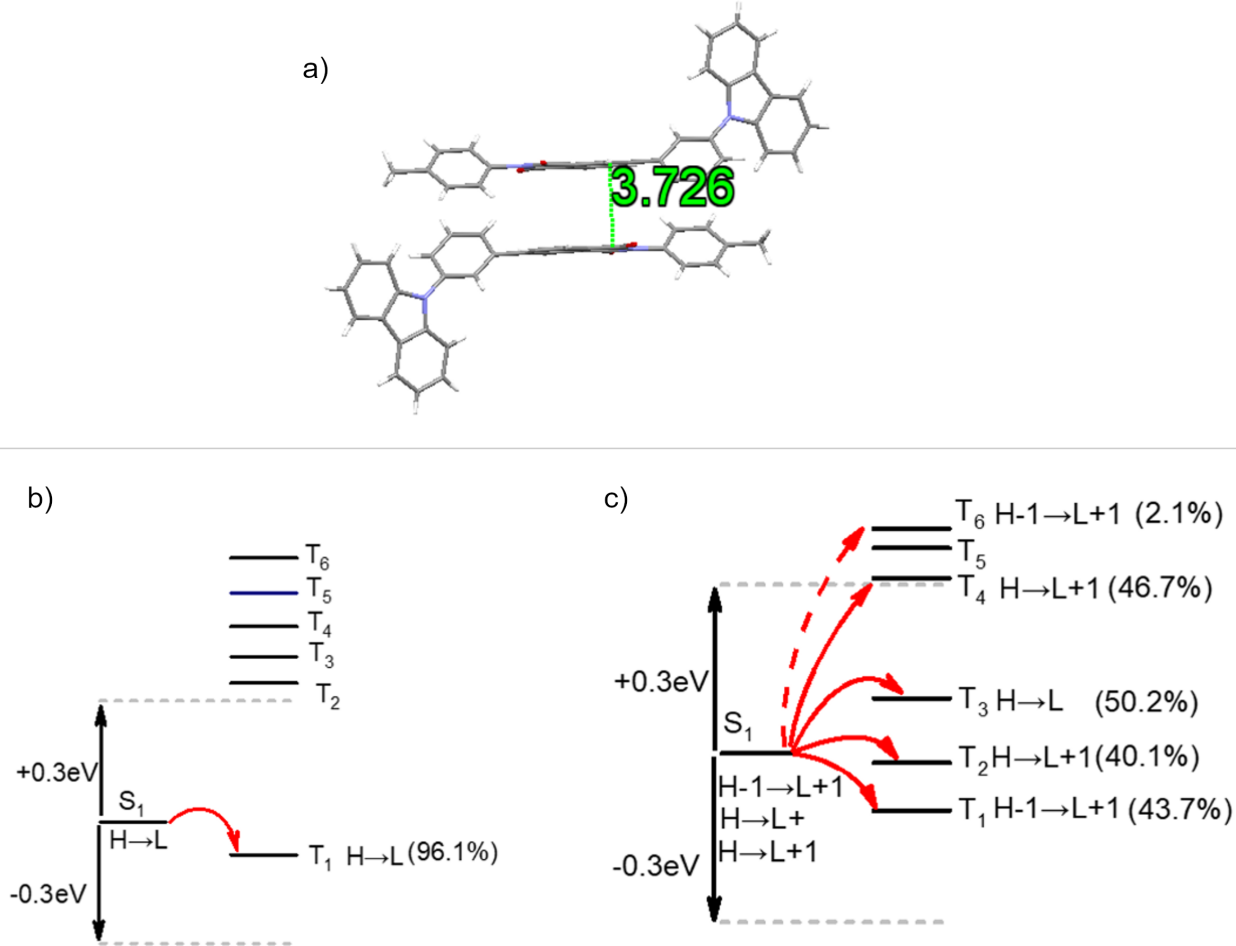
a) The bimolecular packing arrangement in the crystal of **1** and intersystem crossing pathways of **1** in b) single-molecule model and c) bimolecular model.

To investigate the packing arrangement necessary for the manifestation of RTP in **1**, an in-depth analysis was conducted on both the molecular structure and the packing arrangement in single crystals of **1**. As depicted in Figure 6a, the crystal structure of **1** reveals the presence of significant π–π interactions between adjacent molecules. These interactions are crucial for stabilizing the excitons within the crystal, thus facilitating the generation of room-temperature phosphorescence. The presence of these intermolecular π–π interactions enhances the efficiency of phosphorescence by providing a stable environment for the triplet excitons, which are otherwise prone to non-radiative decay in the absence of such stabilizing forces. The phosphorescence spectra recorded from individual molecules of **1** in the crystalline state differ markedly from those reported in the literature for carbazole and phthalimide monomers. These differences can be attributed to the unique molecular packing and the intermolecular interactions within the crystal structure of **1**, which alter the photophysical properties compared to the isolated monomers. In contrast to traditional light-emitting (LE) processes, which typically exhibit a fine structure due to vibrational transitions and other molecular interactions, the single-molecule phosphorescence of **1**, as observed in this study, does not exhibit such fine peaks. This lack of fine structure suggests that the observed emission originates from charge-transfer states, rather than from the typical localized excited states seen in LE emission. At room temperature, the emission wavelength observed for **1** is red-shifted compared to that of single-molecule phosphorescence. This red shift is indicative of the influence of intermolecular π–π interactions, which stabilize the triplet excitons and shift the emission to lower energies. The molecular packing, facilitated by the π–π interactions between adjacent molecules, plays a key role in the stabilization of the triplet state, which in turn contributes to the observed room-temperature phosphorescence. This finding underscores the critical role that molecular arrangement and packing have in governing the photophysical properties of **1**, particularly its ability to exhibit RTP at room temperature.

TD-DFT (time-dependent density functional theory) calculations were carried out to explore and analyze the theoretical structure and electronic properties of the compound. These calculations provided valuable insights into the behavior of the compound in different molecular environments and helped to understand the underlying factors governing its photophysical properties. As depicted in Figure 6b and c, the results from the single-molecule model show that there is only one pathway for intersystem crossing (ISC), which is a relatively simple and straightforward process. However, when considering the bimolecular model, a much more complex set of ISC pathways emerges, indicating that the interactions between adjacent molecules introduce additional pathways for the triplet-to-singlet transition.

The comparison between the single-molecule and bimolecular models reveals a significant difference in the ISC pathways. The bimolecular model exhibits a much greater number of ISC pathways, highlighting the importance of electronic coupling between adjacent molecules in the compound. This electronic coupling not only increases the number of possible ISC pathways but also plays a crucial role in facilitating the efficient intersystem crossing process. This suggests that the intermolecular interactions in the solid-state environment are key factors in controlling the efficiency of the triplet exciton utilization.

Furthermore, the π–π interactions between the molecules in the compound are particularly important. These interactions effectively suppress the torsional motion of the excited-state molecules, which is a key factor in preventing non-radiative decay pathways that would otherwise lead to energy loss. The suppression of these unwanted decay processes further promotes the stability of the excited-state and allows for efficient emission. The π–π interactions between the two molecules in the compound are thus crucial for stabilizing the triplet excitons, which leads to the observed RTP. This finding underscores the importance of molecular packing and intermolecular interactions in achieving efficient phosphorescence at room temperature, making this compound a promising candidate for applications in optoelectronic devices that require stable and efficient RTP behavior.

**Figure 7:**
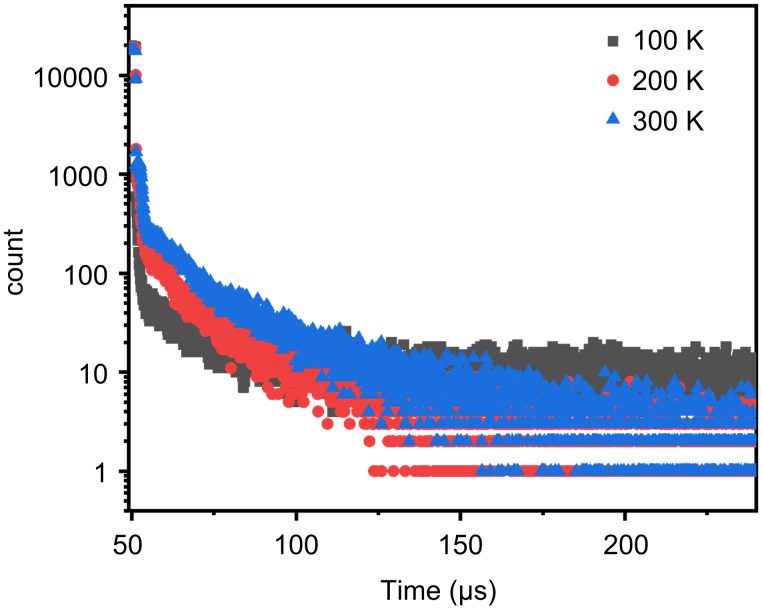
Photoluminescence lifetime of vacuum-deposited **1** films at different temperatures (excitation wavelength: 365 nm).

The films of **1** that were prepared under vacuum thermal evaporation conditions are found to be amorphous in nature, and as such, they do not display any distinguishable RTP at ambient temperature. The absence of RTP in these films is likely due to the lack of an ordered, crystalline structure that is necessary for triplet excitons to undergo the radiative transition typically seen in phosphorescence. To further investigate the photophysical properties of these films, temperature-dependent lifetime measurements were conducted, as shown in Figure 7. These measurements revealed a significant increase in the long-lived (delayed) component of the emission as the temperature was raised. This behavior is indicative of a TADF process, as the total lifetime of the films exhibited typical thermal activation characteristics, further confirming the occurrence of TADF in the amorphous films.

From the previously discussed small Δ*E*_ST_, it can be reasonably inferred that, in the amorphous state, intermolecular π–π stacking interactions are significantly suppressed. This suppression of π–π interactions, along with a more disordered molecular conformation and microenvironment, makes it difficult to establish effective rigid constraints necessary for triplet formation. Additionally, the lack of molecular order in the amorphous state hinders the enhancement of spin–orbit coupling conditions, which are crucial for promoting phosphorescence. As a result, the efficiency of phosphorescence in the triplet state is severely limited in these amorphous films. On the other hand, the small Δ*E*_ST_ plays a critical role in facilitating the RISC process, as it lowers the thermal activation barrier between the S_1_ and T_1_ states. This small energy gap makes it easier for the T_1_ to upconvert to the S_1_ through the RISC process, resulting in the release of energy in the form of delayed fluorescence. Consequently, the delayed fluorescence component in the films of **1** increases with temperature, with the delayed emission being primarily dominated by TADF, rather than phosphorescence. This finding highlights the critical role of the molecular packing, energy gap, and spin–orbit coupling in determining the emission behavior of organic materials, particularly in amorphous states where the TADF process becomes the dominant emission pathway under thermal activation.

**Figure 8:**
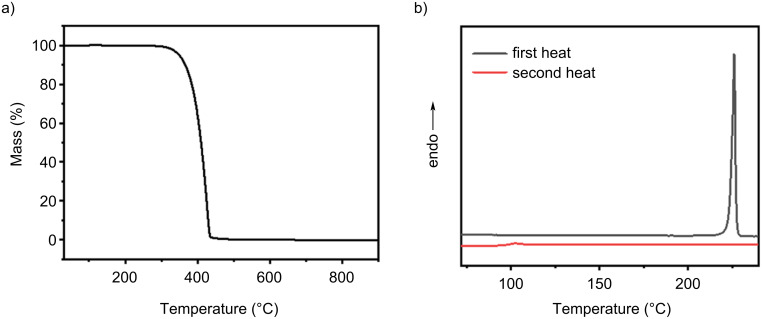
a) TG curve of **1** and b) DSC curve of **1**.

The thermal stability of compound **1** was evaluated using thermogravimetric analysis (TGA) and differential scanning calorimetry (DSC). The results show that **1** exhibits excellent thermal stability. As shown in Figure 8, TGA measurements indicate a 5% weight-loss temperature of 354 °C, suggesting that the material undergoes significant decomposition at temperatures much higher than those typically encountered during device processing and operation, providing a substantial thermal margin. DSC analysis reveals a glass-transition temperature (*T*_g_) of 106 °C and a melting temperature (*T*_m_) of 226 °C. The relatively high *T*_g_ indicates that the material remains in a glassy state from room temperature to moderate temperatures, with limited molecular motion, making it resistant to thermal-induced morphological relaxation and performance drift. The *T*_m_ establishes the upper temperature limit for the solid-to-melt transition of the material. The excellent thermal stability of compound **1** makes it suitable for the fabrication of OLEDs using vacuum deposition and ensures the long-term reliability of the devices.

Due to the excellent TADF properties and good thermal stability of compound **1** in the film state, it can be used as an efficient emissive layer for OLED fabrication. The device structure used was ITO/PEDOT:PSS (40 nm)/mCP (20 nm)/CBP:1 (15 nm, 12% doping)/TPBi (40 nm)/LiF (1 nm)/Al (120 nm). In this structure, PEDOT:PSS, a conductive polymer serves as the hole-injection layer, LiF acts as the electron-injection layer; mCP – 9,9'-(1,3-phenyl)-2-9*H*-carbazole, is the hole-transport material, TPBi – 1,3,5-tris(1-phenyl-1*H*-benzoimidazol-2-yl)benzene, is the electron-transport material, and CBP – 4,4′-*N*,*N*′-dicarbazolebiphenyl is used as the host material mixed with **1** to form the emissive layer.

**Figure 9:**
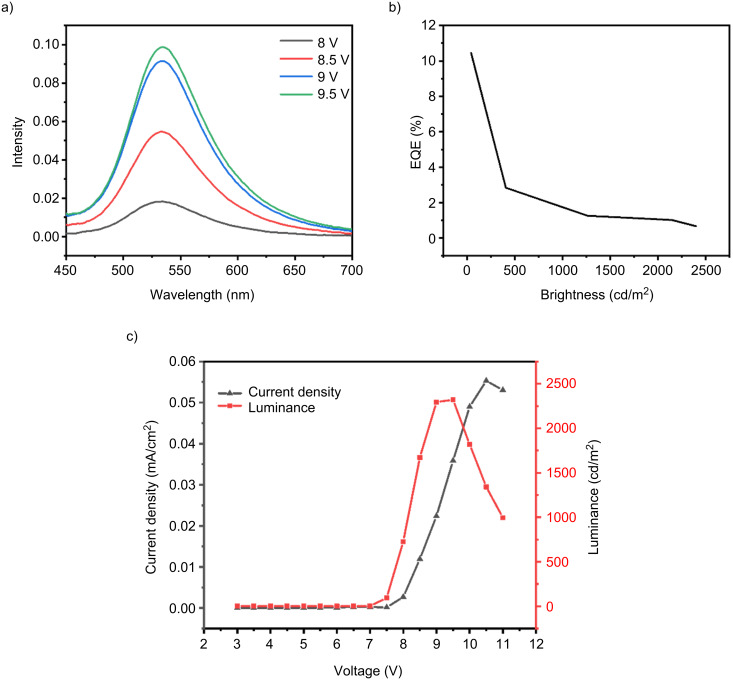
a) Electroluminescence spectrum of the device with **1** as the emissive layer, b) relationship between external quantum efficiency and brightness and c) current density–voltage–luminance characteristics of the OLED device based on **1** measured under ambient conditions.

As illustrated in Figure 9a, the electroluminescence peak of the fabricated OLED is observed at 533 nm. This peak position corresponds to the wavelength at which the device emits its maximum electroluminescent output, showcasing the characteristic emission of the material. Notably, the electroluminescence spectrum of the device remains stable across a range of applied voltages, indicating that the device maintains consistent performance and does not exhibit any significant shift or degradation in emission color as the voltage increases. This behavior is a strong indication of the stability of the OLED, which is a crucial factor for ensuring reliable performance over time, especially for applications that require long-term operation. The current density–voltage–luminance characteristics of the OLED device were measured to evaluate the electrical and optical performance. As shown in Figure 9c, the device exhibits typical J–V–L behavior under electrical bias, confirming the successful operation of the electroluminescent device.

As shown in Figure 9b, the maximum external quantum efficiency (EQE) of the device was measured to be 10.45%, which is a significant achievement as it exceeds the traditional limit of 5% EQE typically observed in many organic light-emitting devices. This higher EQE value demonstrates that the device is effectively utilizing triplet excitons, which is a crucial component for improving the overall efficiency of OLEDs. The ability to achieve such a high EQE is further evidence that the device benefits from the efficient utilization of triplet excitons through processes such as TADF. This result confirms the presence of TADF properties in the fabricated OLED, which are essential for enhancing the performance of the device by enabling more efficient light emission from both singlet and triplet states. The stable electroluminescence spectrum and the high external quantum efficiency observed in the device not only indicate the stability and reliability of the OLED but also highlight the effective exploitation of triplet excitons, confirming the presence of TADF characteristics in the device. These findings provide strong evidence of the successful integration of TADF properties into the OLED, which is essential for developing next-generation high-performance organic light-emitting devices with improved efficiency and stability.

Phthalimide–carbazole (aromatic-imide) donor–acceptor systems have been widely explored as purely organic emitters, where molecular linkage/geometry and intermolecular interactions strongly influence the balance between charge-transfer character and local excited states, and thus the propensity for TADF or RTP [37–39]. In comparison with representative reported phthalimides–carbazole analogues that primarily focus on TADF performance in amorphous/doped films, our study emphasizes a configuration–packing synergy that enables integrated crystalline-state RTP and amorphous-state TADF within the same molecular framework. This comparison highlights how processing-controlled solid-state organization can switch the emissive pathways and provides additional design guidelines for dual-mode emissive materials.

## Conclusion

This study demonstrates that the solid-state packing arrangement determines the triplet exciton utilization pathway of compound **1**. When the system exhibits moderate, ordered π–π stacking and conformational freezing, spin–orbit coupling is enhanced, and both intra- and intermolecular non-radiative pathways are suppressed. This enables the forbidden T_1_→S_0_ transition to manifest at room temperature, resulting in stable, visible phosphorescence. In contrast, in a disordered/amorphous environment without π–π stacking, the lack of rigid confinement and molecular coupling makes it difficult to satisfy the conditions required for phosphorescence. The small Δ*E*_ST_ in the molecule, however, promotes easier RISC to the S_1_ state, leading to TADF domination. In other words, "ordered π–π stacking favors phosphorescence, while the absence of π–π stacking facilitates TADF" forms the core principle governing triplet exciton diversion and regulation in this molecular system.

## Experimental

### Materials

4-Bromophthalic anhydride (**2**), 4-methylaniline (*p*-toluidine, **3**), and tetrakis(triphenylphosphine)palladium(0) were purchased from Aladdin Biochemical Technology Co., Ltd.; 3-(9*H*-carbazol-9-yl)phenylboronic acid (**5**) was obtained from Sukailu Co., Ltd.; glacial acetic acid, anhydrous sodium sulfate, dichloromethane, *n*-hexane, ethanol, chloroform, and tetrahydrofuran were supplied by Guangzhou Chemical Reagents Co., Ltd. All reagents were used as received without further purification.

### Instruments and measurements

Low-resolution mass spectra were recorded on a DSQ electron-impact (EI) mass spectrometer. High-resolution mass spectra (HRMS) were acquired on a Thermo MAT95XP instrument using fast atom bombardment (FAB) ionization. ^1^H and ^13^C NMR spectra were recorded on a Bruker Avance III 500HD superconducting NMR spectrometer using DMSO-*d*_6_ as the solvent and tetramethylsilane (TMS) as the internal standard. Single crystals suitable for X-ray diffraction analysis were grown from a dichloromethane/*n*-hexane solvent system; diffraction data were collected on a Bruker SMART 1000 CCD system and structures were solved using Olex software. UV–vis absorption spectra of dilute solutions were measured on a Hitachi U-3900 UV–vis spectrophotometer with the corresponding solvent as the blank. Solution-state photoluminescence spectra were recorded on a Shimadzu RF-5301PC fluorescence spectrophotometer. Powder-state photoluminescence and room-temperature phosphorescence spectra were collected with an Ocean Optics QE Pro spectrometer. Fluorescence lifetimes were measured on a HORIBA Jobin Yvon modular fluorescence spectrometer. Thermogravimetric analysis (TGA) was performed on a Shimadzu TGA-50 analyzer. Differential scanning calorimetry (DSC) was carried out on a NETZSCH DSC-204 instrument. All calculations were carried out using Gaussian 09. Ground-state geometries were optimized by DFT at the B3LYP/6-31G(d) level. Frequency calculations at the same level confirmed that the optimized structures correspond to true minima (no imaginary frequencies). Vertical excitation energies were computed by TD-DFT at the B3LYP/6-31G(d) level based on the optimized geometries. Electroluminescent devices were fabricated with a conventional multilayer OLED structure using ITO-coated glass substrates as the anode. Prior to deposition, the substrates were cleaned by standard procedures. The organic layers, including the **1**-based emitting layer, were sequentially deposited by thermal evaporation under high vacuum conditions. A metal cathode was subsequently deposited to complete the devices. The EL spectra were measured under ambient conditions.

### Synthesis

**Scheme 1:**
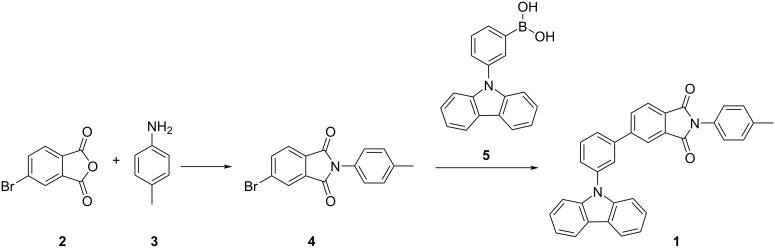
Synthetic pathway to **1** via imide formation and Suzuki–Miyaura cross-coupling.

The synthetic route to **1** is shown in Scheme 1 and comprises imide formation followed by a Suzuki–Miyaura cross coupling.

#### Synthesis of 5-bromo-2-(*p*-tolyl)isoindoline-1,3-dione (PI-Br, 4)

In the first step, **2** (2.00 g, 8.81 mmol) was carefully placed into a 250 mL three-necked round-bottomed flask, ensuring the proper amount of space for the reaction. It was then dissolved in 100 mL of glacial acetic acid, which served as the solvent. To create an inert atmosphere and prevent any unwanted reactions with oxygen, the solution was purged with nitrogen gas for 10 minutes. After this, *p*-toluidine (**3**, 1.13 g, 10.57 mmol) was added slowly to the flask, ensuring thorough mixing of the reactants. The reaction mixture was then heated to reflux overnight, allowing for the completion of the reaction. Upon reaching the desired reaction time, the mixture was cooled to room temperature, and the solvent was removed by pouring the solution into 300 mL of water. This caused the formation of a precipitate, which was collected by suction filtration. The collected solid was dried to remove any remaining moisture. To ensure the product was of high purity, the dried crude product was purified by column chromatography using a solvent mixture of dichloromethane and *n*-hexane in a 1:2 volume ratio. The purified product **4** was obtained as a white solid in a yield of 2.53 g (91%).

#### Synthesis of 5-(3-(9*H*-carbazol-9-yl)phenyl)-2-(*p*-tolyl)isoindoline-1,3-dione (1)

For the synthesis of compound **1**, 5-bromo-2-(*p*-tolyl)isoindoline-1,3-dione (**4**, 1.00 g, 3.16 mmol) was first dissolved in 80 mL of tetrahydrofuran (THF) in a 250 mL three-necked round-bottomed flask. The solution was again purged with nitrogen gas for 30 minutes to create an inert atmosphere and ensure that no moisture or oxygen interfered with the reaction. Following this, 3-(9*H*-carbazol-9-yl)phenylboronic acid (**5**, 1.09 g, 3.80 mmol) was added to the flask. The mixture was stirred for 5 minutes to allow proper mixing. To facilitate the Suzuki coupling reaction, an aqueous solution of potassium carbonate (K_2_CO_3_) with a concentration of 1 g/mL (3 mL) was added. Subsequently, tetrakis(triphenylphosphine)palladium(0) (0.07 g, 0.06 mmol) was introduced to the reaction mixture to promote the coupling reaction. The mixture was then heated to reflux and maintained at this temperature for an overnight period to ensure the reaction proceeded to completion. After the reaction was allowed to cool to room temperature, the mixture was extracted with dichloromethane (3 × 30 mL), which helped separating the organic compounds from the aqueous phase. The combined organic layers were dried over anhydrous sodium sulfate (Na_2_SO_4_) to remove any traces of water. The dried solution was then filtered to remove the drying agent and concentrated under reduced pressure to remove the solvent. The resulting crude product was purified by column chromatography using a 1:2 volume ratio of dichloromethane and *n*-hexane. The final pure product, 5-(3-(9*H*-carbazol-9-yl)phenyl)-2-(*p*-tolyl)isoindoline-1,3-dione (**1**), was obtained as a solid in a yield of 1.36 g (90%). ^1^H NMR (500 MHz, DMSO-*d*_6_) δ 8.34 (s, 1H), 8.31(d, 1H), 8.27 (d, 2H), 8.13 (s, 1H), 8.07–7.98 (m, 2H), 7.84 (t, 1H), 7.74 (d, 1H), 7.53–7.42 (m, 4H), 7.33 (s, 4H), 7.30 (d, 2H), 2.37 (s, 3H); ^13^C NMR (126 MHz, DMSO-*d*_6_) δ 167.23, 145.94, 140.78, 140.61, 138.29, 133.75, 133.11, 132.41, 131.58, 131.15, 129.34, 128.56, 127.85, 127.57, 127.08, 126.82, 126.05, 124.56, 123.30, 122.35, 121.02, 120.64, 110.24; EIMS (*m/z*): [M]^+^ calcd for C_33_H_22_N_2_O_2_, 478.1681; found, 478.1682.

## Supporting Information

File 1 Copies of spectra, crystal data and structure refinement table for compound **1**.

File 2 Crystallographic information file for compound **1**.

File 3 Checkcif file for compound **1**.

## Data Availability

All data that supports the findings of this study is available in the published article and/or the supporting information of this article.
