# Supplementary material for: Configuration–packing synergy enabling integrated crystalline-state RTP and amorphous-state TADF
Source: Beilstein J Org Chem. 2026 Feb 2;22:224–36. doi: 10.3762/bjoc.22.16 (PMC12884550; doi:10.3762/bjoc.22.16)
Supplement: File 1 — Copies of spectra, crystal data and structure refinement table for compound 1. [file Beilstein_J_Org_Chem-22-224-s001.pdf]

BEILSTEIN JOURNAL OF ORGANIC CHEMISTRY

Supporting Information

for

Configuration–packing synergy enabling integrated crystalline-state RTP and amorphous-state TADF

Ruiyan Wang and Yunan Wu

Beilstein J. Org. Chem. **2026,** *22,* 224–236. doi:10.3762/bjoc.22.16

Copies of spectra, crystal data and structure refinement table for compound 1

License and Terms: This is a supporting information file under the terms of the Creative Commons Attribution License (https://creativecommons.org/licenses/by/4.0). Please note that the reuse, redistribution and reproduction in particular requires that the author(s) and source are credited and that individual graphics may be subject to special legal provisions.
The license is subject to the *Beilstein Journal of Organic Chemistry* terms and conditions: (https://www.beilstein-journals.org/bjoc/terms)

Supporting Information

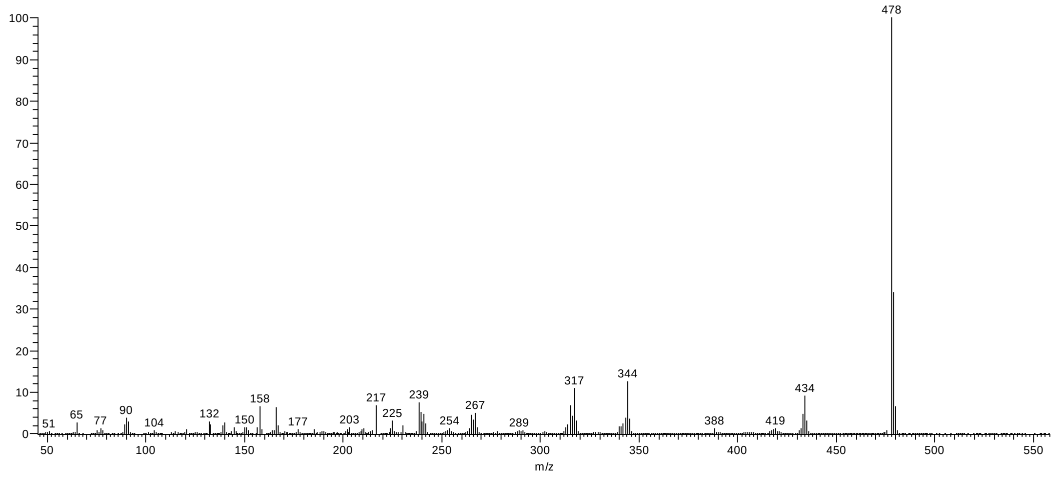

Figure S1. The mass spectrum of compound **1**

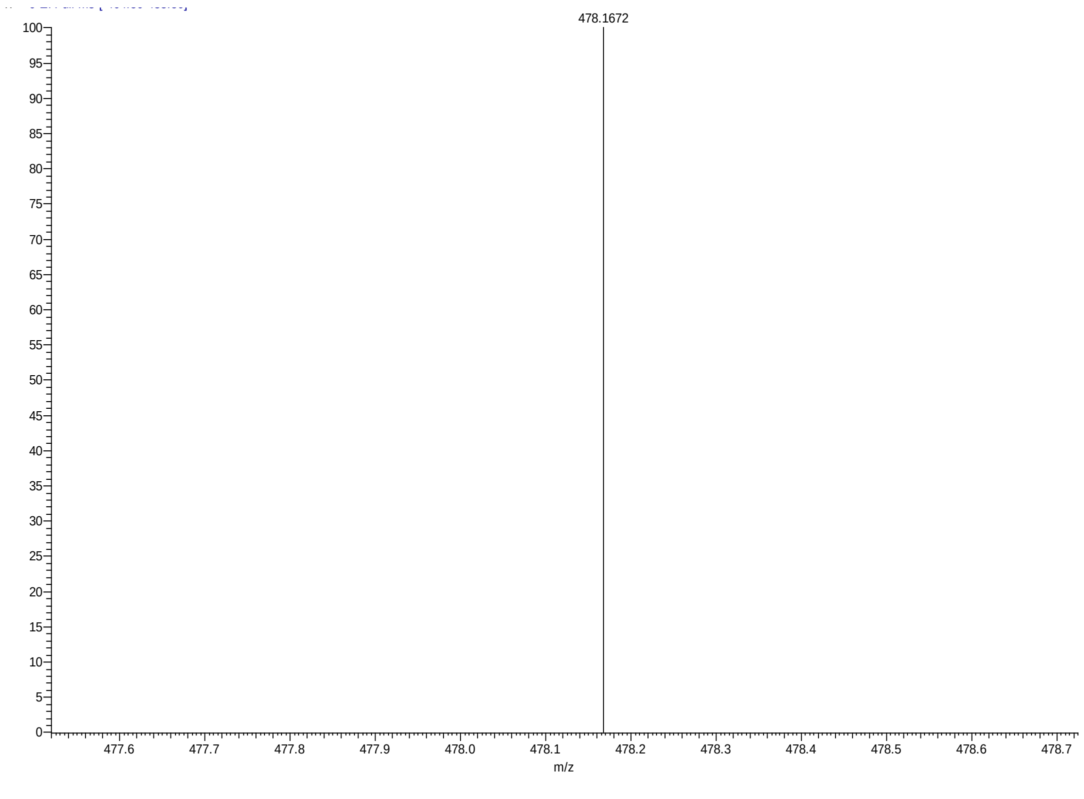

Figure S2. The high resolution mass spectrum of compound **1**

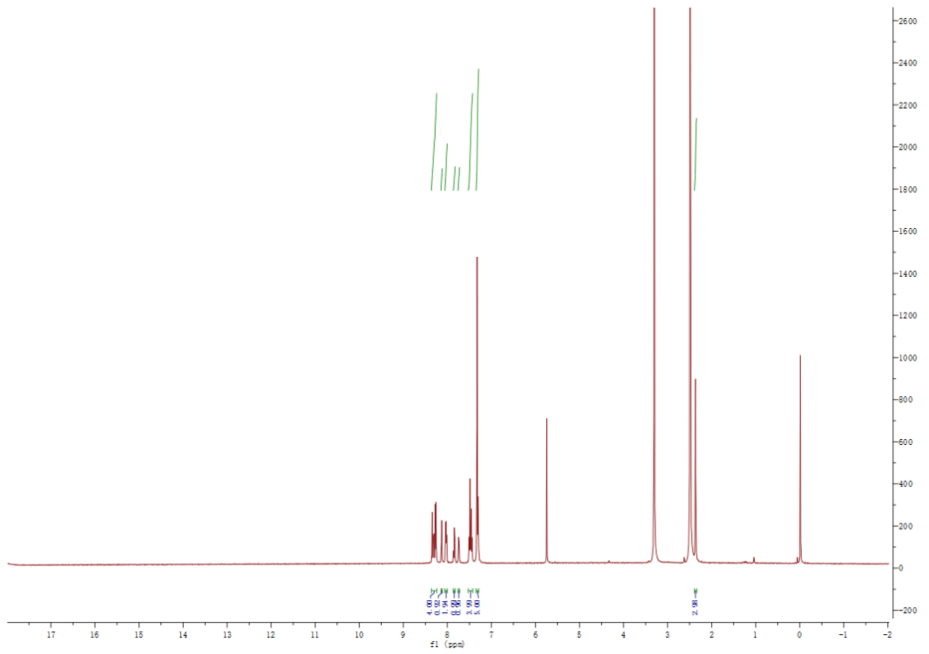

Figure S3. The ^{1}H NMR spectrum of compound **1** in DMSO-d$_6$.

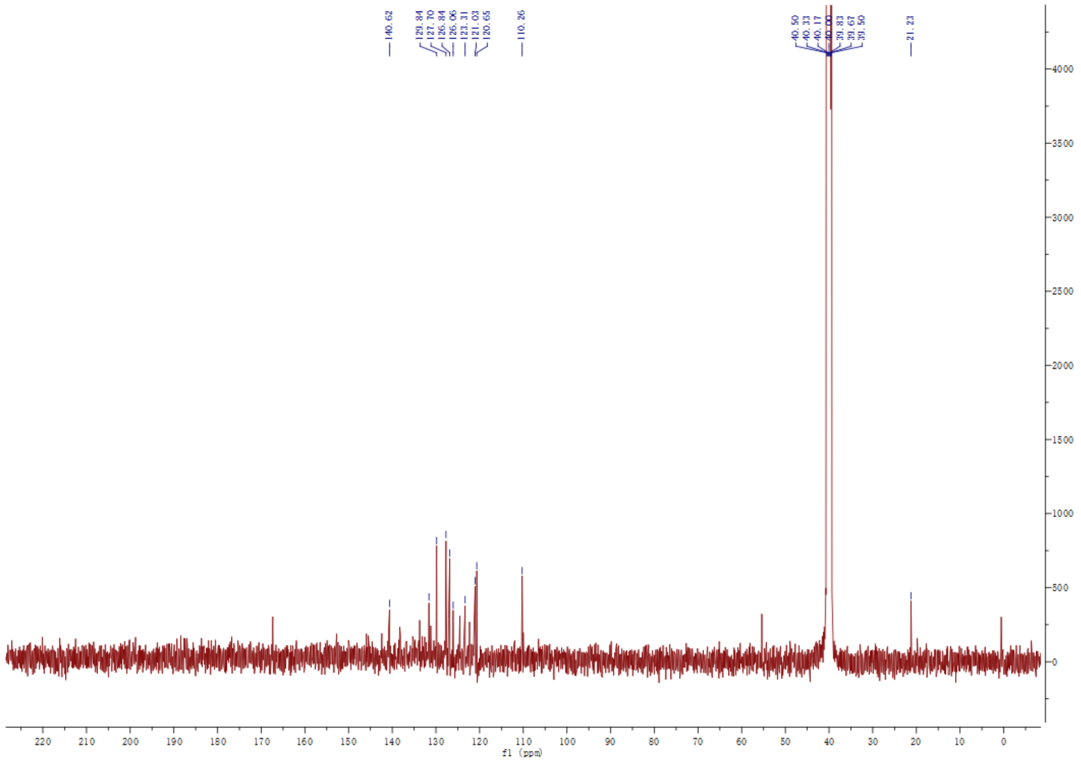

Figure S4. The ^{1}C NMR spectrum of compound **1** in DMSO-d$_6$.

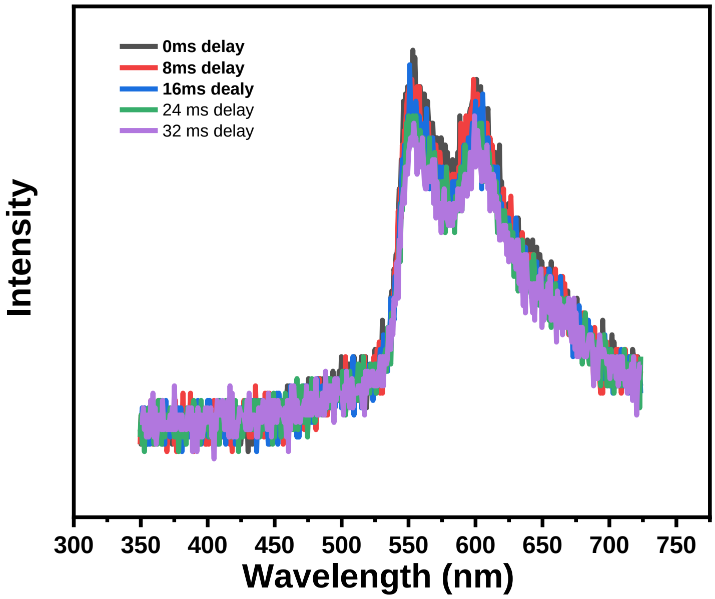

Figure S5. The TRES spectra of 1 powder.

Table S1. The lifetime of **1** powder

Temperature (K)	Lifetime (s)
100	1.13
200	1.12
300	0.47

Table S2. The lifetime of **1** film

Temperature(K)	τ_1(μs)	B_1(%)	τ_2(μs)	B_2(%)	Lifetime(μs)
100	0.446	30.86	14.69	69.14	10.3
200	1.334	31.85	19.9	68.15	14
300	0.854	36.28	16.48	63.72	16.48

Table S3. Crystal data and structure refinement for **1**

Compound	**1**
Empirical formula	$C_{33}H_{22}N_2O_2$
Formula weight	478.52
Temperature/K	220.00(10)
Crystal system	monoclinic
Space group	$P2_1/c$
a/Å	8.6894(2)
b/Å	35.8672(5)
c/Å	8.10020(10)
α/°	90
β/°	107.351(2)
γ/°	90
Volume/Å^3	2409.67(8)
Z	4
ρ_{calc}g/cm^3	1.319
μ/mm^{-1}	0.653
F(000)	1000.0
Crystal size/mm^3	0.23 × 0.15 × 0.04
Radiation	CuKα (λ = 1.54184)
2Θ range for data collection/°	9.864 to 146.646
Index ranges	$-10 \leq h \leq 9$, $-43 \leq k \leq 40$, $-9 \leq l \leq 6$
Reflections collected	7798
Independent reflections	4617 [R_{int} = 0.0301, R_{sigma} = 0.0400]
Data/restraints/parameters	4617/0/335
Goodness-of-fit on F^2	0.997
Final R indexes [I>=2σ (I)]	R_1 = 0.0489, wR_2 = 0.1351
Final R indexes [all data]	R_1 = 0.0544, wR_2 = 0.1427
