# Supplementary material for: Configuration–packing synergy enabling integrated crystalline-state RTP and amorphous-state TADF
Source: Beilstein J Org Chem. 2026 Feb 2;22:224–36. doi: 10.3762/bjoc.22.16 (PMC12884550; doi:10.3762/bjoc.22.16)

BEILSTEIN JOURNAL OF ORGANIC CHEMISTRY

Supporting Information

for

Configuration–packing synergy enabling integrated crystalline-state RTP and amorphous-state TADF

Ruiyan Wang and Yunan Wu

Beilstein J. Org. Chem. **2026,** *22,* 224–236. doi:10.3762/bjoc.22.16

Checkcif file for compound 1

License and Terms: This is a supporting information file under the terms of the Creative Commons Attribution License (https://creativecommons.org/licenses/by/4.0). Please note that the reuse, redistribution and reproduction in particular requires that the author(s) and source are credited and that individual graphics may be subject to special legal provisions.
The license is subject to the *Beilstein Journal of Organic Chemistry* terms and conditions: (https://www.beilstein-journals.org/bjoc/terms)

checkCIF/PLATON report

Structure factors have been supplied for datablock(s) 19090503

THIS REPORT IS FOR GUIDANCE ONLY. IF USED AS PART OF A REVIEW PROCEDURE FOR PUBLICATION, IT SHOULD NOT REPLACE THE EXPERTISE OF AN EXPERIENCED CRYSTALLOGRAPHIC REFEREE.

No syntax errors found. CIF dictionary Interpreting this report

Datablock: 19090503

```
Bond precision:   C-C = 0.0023 A          Wavelength=1.54184

Cell:             a=8.6894(2)     b=35.8672(5)        c=8.1002(1)
                  alpha=90        beta=107.351(2)     gamma=90
Temperature:      220 K

                 Calculated                   Reported
Volume           2409.67(8)                   2409.67(8)
Space group      P 21/c                       P 1 21/c 1
Hall group       -P 2ybc                      -P 2ybc
Moiety formula   C33 H22 N2 O2                C33 H22 N2 O2
Sum formula      C33 H22 N2 O2                C33 H22 N2 O2
Mr               478.53                       478.52
Dx,g cm-3        1.319                        1.319
Z                4                            4
Mu (mm-1)        0.653                        0.653
F000             1000.0                       1000.0
F000'            1002.87
h,k,lmax         10,44,10                     10,43,9
Nref             4831                         4617
Tmin,Tmax        0.889,0.974                  0.297,1.000
Tmin'            0.861

Correction method= # Reported T Limits: Tmin=0.297 Tmax=1.000
AbsCorr = MULTI-SCAN
```

```
Data completeness= 0.956           Theta(max)= 73.323

R(reflections)= 0.0489( 4070)                              wR2(reflections)=
                                                           0.1427( 4617)
S = 0.997                    Npar= 335
```

The following ALERTS were generated. Each ALERT has the format
test-name_ALERT_alert-type_alert-level.
Click on the hyperlinks for more details of the test.

Alert level C

```
PLAT906_ALERT_3_C Large K Value in the Analysis of Variance ......      2.116 Check
PLAT911_ALERT_3_C Missing FCF Refl Between Thmin & STh/L=    0.600         48 Report
                1  0  0,   2  0  0,   3  0  0,   0 20  0,   0 22  0,   0 24  0,
                6 34  0,   2  1  1,   0 22  1,  -1 23  1,   0 23  1,  -1 24  1,
                0 24  1,   0  0  2,   1  0  2,   2  0  2,   0  1  2,   0 22  2,
              -10  1  5, -10  2  5,   4  0  6,   5  0  6,   5  1  6,   5  2  6,
                5  3  6,  -9 11  6,  -9 12  6,  -9 13  6,  -9 14  6,  -8 21  6,
                   (   18 More Missing: see the .ckf listing file)
```

Alert level G

```
PLAT720_ALERT_4_G Number of Unusual/Non-Standard Labels ..........         59 Note
              O001    O002    N003    N004    C005    C006    C007    C008
              C009    H009    C00A    C00B    C00C    C00D    H00D    C00E
              C00F    H00F    C00G    H00G    C00H    C00I    H00I    C00J
              H00J    C00K    H00K    C00L    H00L    C00M    H00M    C00N
              C00O    C00P    H00P    C00Q    C00R    C00S    H00S    C00T
              H00T    C00U    H00U    C00V    H00V    C00W    H00W    C00X
              H00X    C00Y    H00Y    C00Z    H00A    H00B    H00C    C010
              H010    C011    H011
PLAT910_ALERT_3_G Missing FCF Reflection(s) Below Theta(Min)[Deg]=       4.93 Note
                0  2  0,
PLAT912_ALERT_4_G Missing # of FCF Reflections Above STh/L=  0.600        152 Note
PLAT941_ALERT_3_G Average HKL Measurement Multiplicity ..........         1.7 Low
PLAT969_ALERT_5_G The 'Henn et al.' R-Factor-gap value ..........       2.785 Note
              Predicted wR2: Based on SigI**2  5.13 or SHELX Weight 14.32
PLAT978_ALERT_2_G Number C-C Bonds with Positive Residual Density.          16 Info
PLAT992_ALERT_5_G Repd & Actual _reflns_number_gt Values Differ by          2 Check
```

---

0 **ALERT level A** = Most likely a serious problem - resolve or explain
0 **ALERT level B** = A potentially serious problem, consider carefully
2 **ALERT level C** = Check. Ensure it is not caused by an omission or oversight
7 **ALERT level G** = General information/check it is not something unexpected

0 ALERT type 1 CIF construction/syntax error, inconsistent or missing data
1 ALERT type 2 Indicator that the structure model may be wrong or deficient
4 ALERT type 3 Indicator that the structure quality may be low
2 ALERT type 4 Improvement, methodology, query or suggestion
2 ALERT type 5 Informative message, check

It is advisable to attempt to resolve as many as possible of the alerts in all categories. Often the minor alerts point to easily fixed oversights, errors and omissions in your CIF or refinement strategy, so attention to these fine details can be worthwhile. It is up to the individual to critically assess their own results and, if necessary, seek expert advice.

PLATON version of 26/09/2025; check.def file version of 20/09/2025

---

duplicate check

No duplication found

Datablock 19090503 - ellipsoid plot

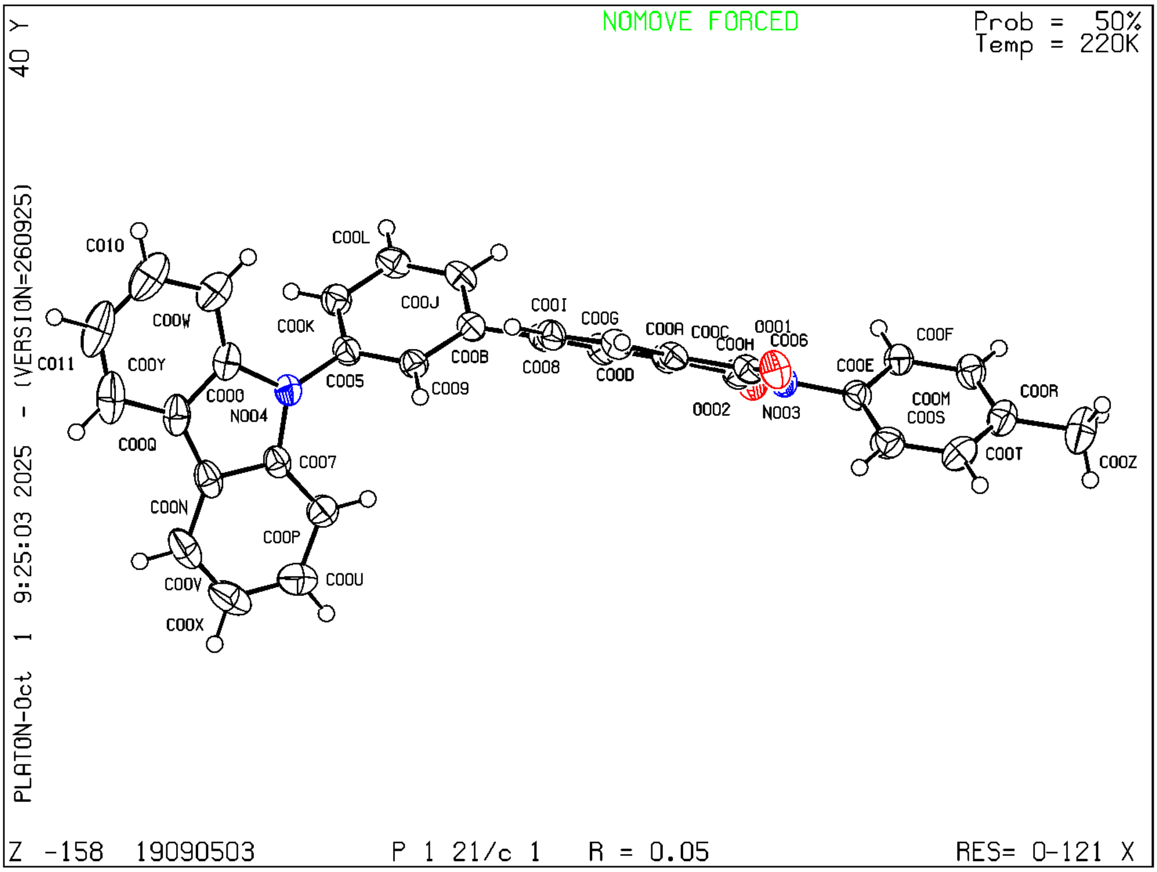

Supplement: File 3 — Checkcif file for compound 1. [file Beilstein_J_Org_Chem-22-224-s003.pdf]
